# Supplementary material for: Heat Treatment Augments Antigen Detection of Dirofilaria immitis in Apparently Healthy Companion Dogs (3.8% to 7.3%): Insights from a Large-Scale Nationwide Survey across the United States
Source: Pathogens. 2024 Jan 6;13(1):56. doi: 10.3390/pathogens13010056 (PMC10818916; doi:10.3390/pathogens13010056)
Supplement: Supplementary file 1 [file pathogens-13-00056-s001.zip › pathogens-2806292-supplementary.pdf]

Table S1. Dogs identified to be HW antigen after heat treatment on sera.

9	Age (year)	Sex	Breed	State
2022	6	Female	German Shepherd Mix	AL
2022	1	Male	Bichon Frise	AL
2022	3	Female	German Shepherd	FL
2022	6	Female	Boxer	FL
2022	10	Male	Papillon	MA
2022	0.58	Female	German Shepherd	CA
2022	0.58	Male	German Shepherd	CA
2022	8	Male	German Shepherd	AL
2022	4	Male	Labrador Retriever Mix	FL
2022	1	Male	Doberman Pinscher	CA
2022	4	Male	Yorkshire Terrier	CA
2022	13	Female	Portuguese Water Dog	TX
2022	0.58	Male	Bichon Frise	CA
2022	0.33	Female	Bichon Frise	CA
2022	11	Female	Labrador Retriever	AL
2022	9	Male	Chihuahua Mix	HI
2022	2	Male	Australian Shepherd	GA
2022	1	Female	Labrador Retriever	AL
2022	6	Male	Maltese	CA
2022	3	Male	Pembroke Welsh Corgi	CA
2022	5	Male	German Shepherd Mix	LA
2022	7	Female	Pembroke Welsh Corgi	CA
2021	1.5	Female	Papillon	CA
2021	5	Male	Plott Hound	GA
2021	4.66	Male	Australian Shepherd	HI
2021	2	Female	American Eskimo	CA
2021	16	Male	Golden Retriever	AL
2021	4	Male	Australian Shepherd	GA
2021	2	Male	Golden Retriever	PA
2021	0.83	Male	Shiba Inu	CA
2021	2	Female	Labrador Retriever Mix	VA
2021	11.33	Female	Dachshund Mix	IL
2021	15	Male	Dachshund	PA
2021	6	Female	Japanese Chin	PA
2021	10	Male	Cavalier King Charles Spaniel	PA
2021	3	Male	Rat Terrier	PA
2021	2	Male	Labrador Retriever	PA
2021	13	Male	Toy Poodle	FL
2021	15	Female	Chihuahua Mix	CA
2021	6.5	Male	Cocker Spaniel	WA
2021	4	Male	American Bulldog	PA
2021	7	Female	Poodle Mix	AL
2021	6.5	Female	American Staffordshire Terrier	FL
2021	1	Female	Boxer Mix	CA
2021	12.91	Female	Terrier Mix	PA
2021	1	Female	Chihuahua Mix	HI
2021	5	Female	Chihuahua Mix	HI
2021	10	Female	Yorkshire Terrier	CA
2021	1.25	Female	Scottish Terrier	TX
2021	4	Female	Schnauzer	AL
2021	1	Male	Shiba Inu	NY
2021	0.5	Male	Shih Tzu	NV
2021	0.41	Female	German Shepherd	NV
2021	0.41	Male	Caucasian Mountain Dog	NV
2021	0.33	Male	Alaskan Malamute	NV
2021	8	Female	Dachshund	HI
2021	4	Female	Terrier Mix	CO
2021	4	Female	Labrador Retriever	PA
2021	4	Male	Golden Retriever	PA
2021	0.91	Female	Dachshund	CO

2021	5	Female	Mixed Breed	CA
2021	10	Female	Australian Shepherd	VA
2021	0.83	Female	Miniature Poodle Mix	NY
2021	10	Female	Basset Hound	FL
2021	2	Male	American Eskimo	VA
2021	10	Male	Pomeranian	GA
2021	5	Male	Australian Cattle Dog Mix	NV
2021	0.41	Female	Poodle Mix	CO
2021	11	Male	Mixed Breed	PA
2021	2	Male	Mixed Breed	PA
2021	4	Female	German Shepherd	CO
2021	16	Male	Yorkshire Terrier	VA
2021	9	Female	Labrador Retriever Mix	KY
2021	4	Male	German Shepherd	HI
2021	12	Female	Australian Shepherd	PA
2021	3	Female	Poodle Miniature	HI
2021	0.37	Female	German Shorthair Pointer	HI
2021	1	Female	Aussie doodle	VA
2021	3.5	Male	Pembroke Welsh Corgi Mix	NY
2021	8	Female	Dachshund	HI
2021	10	Male	Yorkshire Terrier	TN
2021	6	Male	Bichon Frise	MA
2021	0.41	Male	Chihuahua Mix	FL
2021	2	Male	Great Pyrenees	PA
2021	8	Female	German Shepherd	IN
2021	1	Female	Pembroke Welsh Corgi	NY
2021	4	Female	Pit Bull	AL
2021	11	Female	French Bulldog	MN
2021	14	Male	Miniature Pinscher	FL
2021	0.58	Male	Mixed Breed	NY
2021	1	Female	Jindo Mix	WA
2021	8	Female	Chihuahua Mix	NY
2021	3	Female	Labrador Retriever	AL
2021	8	Female	Shih Tzu	TX
2021	6	Male	Mixed Breed	WY
2021	1	Male	Golden Retriever	FL
2021	10	Female	Golden Retriever	GA
2021	1	Male	Chihuahua Mix	MS
2021	2	Male	Labrador Retriever	NC
2021	11	Male	Pomeranian	PA
2021	3	Female	Australian Shepherd	TX
2021	5	Female	Border Collie	WA
2021	9	Female	Miniature Pinscher	PA
2021	2.5	Male	Golden Retriever	NC
2021	1.83	Female	Pomeranian	NY
2021	10	Female	Schnauzer	GA
2021	9	Female	Pit Bull Mix	ID
2021	3.5	Male	German Shorthair Pointer	FL
2021	8	Male	Labrador Retriever	IN
2021	7	Female	Miniature Schnauzer	HI
2021	4	Female	Belgian Malinois	FL
2021	2	Female	Labrador Retriever Mix	AK
2021	1	Female	German Shepherd	NV
2021	3	Male	Cardigan Welsh Corgi	PA
2021	10	Male	Havanese	PA
2021	13	Male	Mixed Breed	PA
2021	5	Female	Boston Terrier	PA
2021	1	Female	Maltese	PA
2021	6	Female	Golden Retriever	PA
2021	6.5	Male	Labrador Retriever Mix	FL
2021	3.5	Male	Dachshund	NY
2021	1.41	Male	Maltese	NY
2021	0.91	Female	Chow Chow	GA
2021	0.58	Female	Parson Russell Terrier	PA
2021	2	Male	Chihuahua Mix	VA

2021	0.66	Male	Shepherd Mix	CO
2021	5	Female	Labrador Retriever Mix	NC
2021	5	Female	Poodle	WA
2021	6	Female	German Shepherd Mix	MO
2021	12	Female	Boxer	FL
2021	0.5	Female	Labrador Retriever	NY
2021	0.33	Male	Border Collie	DE
2021	9.58	Female	Cocker Spaniel	GA
2021	12	Male	Miniature Poodle	IL
2021	19	Female	Chihuahua	FL
2021	1	Female	Papillion	AL
2021	11	Female	Mixed Breed	GA
2020	4	Male	Shepherd	AR
2020	2	Female	Pembroke Welsh Corgi	WI
2020	0.75	Female	Dalmatian	UT
2020	8	Female	Miniature Schnauzer	VT
2020	4	Male	Miniature Schnauzer	VA
2020	2	Female	Golden Retriever	OK
2020	8	Female	Labrador Retriever Mix	VA
2020	5	Female	Boxer Mix	FL
2020	7.66	Female	Labrador Retriever Mix	AL
2020	9.33	Male	Labrador Retriever Mix	AL
2020	3.16	Male	Labrador Retriever Mix	AL
2020	0.91	Male	Siberian Husky	NY
2020	3	Male	French Bulldog	MD
2020	4.5	Male	Poodle	CT
2020	0.66	Female	Poodle	TN
2020	0.41	Female	Boston Terrier Mix	TX
2020	11	Male	Cocker Spaniel	WA
2020	6	Male	Australian Shepherd	KY
2020	12	Female	Beagle Mix	TN
2020	0.5	Female	French Bulldog	MS
2020	2	Male	Golden Retriever	NE
2020	2	Female	Labradoodle	MI
2020	4	Female	Yorkshire Terrier	TX
2020	0.33	Male	Goldendoodle	TX
2020	5	Male	American Bulldog	ID
2020	2	Female	Yorkshire Terrier	TX
2020	0.75	Female	Brittany Spaniel	AL
2020	0.5	Female	Australian Shepherd	WA
2020	7	Female	Border Collie Mix	TX
2020	11	Male	Pug	MT
2020	2	Male	Siberian Husky	IL
2020	1	Female	Pembroke Welsh Corgi	MO
2020	1	Female	Pomeranian	WA
2020	1.5	Female	Dalmatian	TX
2020	6.75	Female	Jack Russell Terrier	IL
2020	6	Female	Toy Poodle	MS
2020	10	Male	Chihuahua	MS
2020	2	Male	Poodle Mix	MA
2020	9	Male	Dachshund Mix	TX
2020	2.08	Female	Hound	WA
2020	0.5	Male	Mixed Breed	OK
2020	7	Female	Labrador Retriever Mix	TN
2020	8	Female	Border Collie	TN
2020	13.5	Female	Jack Russell Terrier	TN
2020	7	Female	Siberian Husky	WA
2020	3	Female	Great Dane	WA
2020	11	Female	Poodle	TN
2020	3	Male	Rat Terrier	IL
2020	4	Female	Siberian Husky	NC
2020	1	Male	Goldendoodle	TX
2020	2	Male	Chihuahua	TN
2020	0.91	Male	Rottweiler	NC
2020	13	Male	Miniature Schnauzer	OK

2020	9	Female	Kelpie	ID
2020	1	Female	Great Dane	MO
2020	7	Male	Great Pyrenees Mix	TN
2020	7	Female	Yorkshire Terrier	KY
2019	3	Male	Rat Terrier	IL
2019	8	Male	Shih Tzu	LA
2019	7	Male	Terrier Mix	UT
2019	0.91	Male	Labrador Retriever Mix	MO
2019	2	Female	Blue Lacy	TX
2019	6.16	Male	Labrador Retriever Mix	MS
2019	2	Female	Weimaraner	MO
2019	1.66	Male	American Bully	LA
2019	0.75	Male	Labrador Retriever Mix	MO
2019	2	Male	Beagle Mix	IA
2019	1	Female	German Shepherd Mix	IN
2019	12	Male	Belgian Tervuren	IL
2019	2	Male	Schnauzer	KY
2019	0.91	Male	Shih Tzu Mix	RI
2018	15	Female	Mixed Breed	TN
2018	9	Female Spayed	West Highland White Terrier	KY
2018	2	Female Spayed	Poodle	TX
2018	8	Female	Poodle Miniature	TN
2018	8	Female Spayed	Poodle	IL
2018	NS	Female Spayed	Jack Russell Terrier	KY
2017	5	Female Spayed	Maltese	TN
2017	2	Female Spayed	German Shepherd Mix	TX
2017	4	Male Castrated	Poodle Miniature	KY
2017	9	Male Castrated	Poodle Mix	NJ
2017	11	Female Spayed	Labrador Retriever Mix	TN
2017	8	Male Castrated	Boston Terrier	TN
2017	3	Male Castrated	Goldendoodle	TN
2017	2	Female Spayed	Goldendoodle	TN
2017	9	Male Castrated	Rottweiler	TN
2017	8	Female Spayed	Maltese	TN
2017	14	Male Castrated	Dachshund Mix	KY
2017	9	Female Spayed	Shetland Sheepdog	TN
2017	2	Male Castrated	Chihuahua Mix	NC
2017	1	Female	American Staffordshire Terrier	TN
2017	11	Male Castrated	Husky Mix	TN
2017	13	NS	Shih Tzu	IL
2017	2	Male Castrated	Chihuahua Mix	NC
2017	NS	Female Spayed	Bichon Frise	TN
2017	13	Female Spayed	English Cocker Spaniel	TN
2017	4	Female Spayed	Yorkshire Terrier	KY
2017	4	Female Spayed	Yorkshire Terrier	KY
2017	6	Female Spayed	Labrador Retriever Mix	TN
2017	6	Male Castrated	Labrador Retriever Mix	TN

NS indicates the sex of the dog was not specified.
